# SARCOPENIA: NO CONSENSUS, NO DIAGNOSTIC CRITERIA, AND NO APPROVED INDICATION HOW DID WE GET HERE?

**DOI:** 10.1093/geroni/igae098.1954

**Published:** 2024-12-31

**Authors:** William Evans, Peggy Cawthon

**Affiliations:** Chair; Co-Chair

## Abstract

In addition to the role of skeletal muscle in movement and locomotion, muscle plays a critical role in a broad array of metabolic processes that can contribute to improved health or risk of disease. The age-associated loss of muscle has been termed sarcopenia. Muscle is the primary site of insulin-stimulated glucose disposal and the largest component of basal metabolic rate, directly and indirectly affects bone density, produces myokines with pleiotropic effect on muscle and other tissues including the brain, stores essential amino acids essential for the maintenance of protein synthesis during periods of reduced food intake and stress. As such, not surprisingly deterioration of skeletal muscle health, typically operationalized as decline of muscle mass and muscle strength is both a powerful risk factor and main consequence of chronic diseases, disability, and loss of independence, and it is one of the strongest risk factors for mortality. As a result, the development of pharmacological therapies to increase muscle mass (or prevent loss) an important goal for decades. However, while remarkable advances in the understanding of molecular and cellular regulation of muscle protein metabolism have occurred recently, there are no approved drugs for the treatment of sarcopenia, the loss of skeletal muscle affecting millions of older people The goal of this symposium is to describe the possible reasons for the lack of new and effective pharmacotherapies to treat one of the most important risk factors for age-associated disease and loss of independence.

